# Reduced grid-like theta modulation in schizophrenia

**DOI:** 10.1093/brain/awac416

**Published:** 2022-11-10

**Authors:** Laura Convertino, Daniel Bush, Fanfan Zheng, Rick A Adams, Neil Burgess

**Affiliations:** UCL Institute of Cognitive Neuroscience, University College London, London WC1N 3AZ, UK; Wellcome Centre for Human Neuroimaging, University College London, London WC1N 3AR, UK; Department of Neuroscience, Physiology and Pharmacology, University College London, London WC1E 6BT, UK; School of Nursing, Peking Union Medical College, Chinese Academy of Medical Sciences, Beijing, China; UCL Institute of Cognitive Neuroscience, University College London, London WC1N 3AZ, UK; Wellcome Centre for Human Neuroimaging, University College London, London WC1N 3AR, UK; Division of Psychiatry, University College London, London W1T 7BN, UK; Max Planck-UCL Centre for Computational Psychiatry and Ageing Research, London WC1B 5EH, UK; Centre for Medical Image Computing, Department of Computer Science, University College London, London WC1V 6LJ, UK; UCL Institute of Cognitive Neuroscience, University College London, London WC1N 3AZ, UK; Wellcome Centre for Human Neuroimaging, University College London, London WC1N 3AR, UK; UCL Queen Square Institute of Neurology, University College London, London WC1N 3BG, UK

**Keywords:** schizophrenia, entorhinal cortex, grid cells, spatial memory

## Abstract

The hippocampal formation has been implicated in the pathophysiology of schizophrenia, with patients showing impairments in spatial and relational cognition, structural changes in entorhinal cortex and reduced theta coherence with medial prefrontal cortex. Both the entorhinal cortex and medial prefrontal cortex exhibit a 6-fold (or ‘hexadirectional’) modulation of neural activity during virtual navigation that is indicative of grid cell populations and associated with accurate spatial navigation.

Here, we examined whether these grid-like patterns are disrupted in schizophrenia. We asked 17 participants with diagnoses of schizophrenia and 23 controls (matched for age, sex and IQ) to perform a virtual reality spatial navigation task during magnetoencephalography.

The control group showed stronger 4–10 Hz theta power during movement onset, as well as hexadirectional modulation of theta band oscillatory activity in the right entorhinal cortex whose directional stability across trials correlated with navigational accuracy. This hexadirectional modulation was absent in schizophrenia patients, with a significant difference between groups.

These results suggest that impairments in spatial and relational cognition associated with schizophrenia may arise from disrupted grid firing patterns in entorhinal cortex.

## Teaser

Convertino *et al.* report that people with schizophrenia lack the hexadirectional modulation of theta power in right entorhinal cortex observed in a matched control group. Impairments in spatial and relational cognition associated with schizophrenia may thus arise from disrupted grid firing patterns in entorhinal cortex.

## Introduction

Schizophrenia is characterized by distortion of thoughts and perception including delusions, hallucinations, disorganized or catatonic behaviour and diminished emotional expression or motivation [Diagnostic and Statistical Manual of Mental Disorders. 5th ed. (DSM-5)^1^ ]. Several studies suggest a role for the hippocampal formation in the pathophysiology of schizophrenia.^2–6^ Specifically, patients exhibit structural changes in entorhinal cortex^7–9^ and reduced functional connectivity between the medial temporal lobe (MTL) and medial prefrontal cortex (mPFC).^2,10–13^ The hippocampal formation plays a fundamental role in episodic memory and spatial navigation.^14,15^ Consistent with this, patients with schizophrenia also exhibit impaired performance in a range of spatial navigation tasks.^16–20^

Spatial cognition appears to depend on specialized populations of neurons including grid cells,^21^ originally identified in the rodent medial entorhinal cortex and subsequently found in the human entorhinal cortex and mPFC.^22^ Grid cells exhibit periodic spatial firing fields with 6-fold (or ‘hexadirectional’) rotational symmetry. Grid cells are thought to support accurate spatial navigation^23–25^ and may also contribute to relational memory^26,27^ and the acquisition of structural knowledge.^28^ Hence, we examined whether grid cell activity patterns might be disrupted in schizophrenia.

In rodents, grid cell firing patterns appear to depend on movement-related theta band oscillations.^29–31^ There is also evidence for movement-related theta oscillations in human intracranial local field potentials,^32–34^ particularly during movement initiation.^35^ Hexadirectional modulation of theta band activity, consistent with the presence of grid cell firing patterns, has also been observed in intracranial EEG recordings from the entorhinal cortex during virtual navigation,^36,37^ building on observations of similar patterns in blood oxygen level-dependent (BOLD) signal throughout the default mode network.^38^ We therefore asked participants with a diagnosis of schizophrenia (half of whom were unmedicated) and a matched control group to complete an established spatial navigation task inside a magnetoencephalography (MEG) scanner.^2,39,40^ We then looked for hexadirectional modulation of theta band oscillatory activity during virtual movement.

## Materials and methods

### Participants

This study re-analyses MEG data first presented in Adams *et al*.^2^ The study was approved by the local NHS research ethics board (REF: 17/LO/0027) and all participants gave informed consent. Age, sex, IQ, digit span, handedness and years in education information was collected from all participants. Participants with a schizophrenia diagnosis also completed the Positive and Negative Symptoms Scale,^41^ a saliva recreational drugs test (see Supplementary Table 1 in Adams *et al*.^2^) and documented their medication. To be included, participants must have been educated in English, not be using benzodiazepines or anticonvulsants, have normal (or corrected to normal) vision and be under 60 years old. The patient group was recruited based on DSM-IV criteria for schizophrenia, with 18 participants in total. Patients had no other psychiatric diagnoses, based on the structured clinical interview for DSM-IV-TR axis I disorders.^42^ The control group were recruited to match the age, sex and IQ of the patient group as closely as possible, with 35 participants in total. Controls were excluded if they had history of a psychiatric or neurological condition. In addition, 1 patient and 12 control participants were excluded due to excessive MEG artefacts, interruption of the experiment due to nausea or sleep or loss of fiducial markers. This left 17 patients (14 males) and 23 controls (17 males). All participants were asked not to consume caffeine or smoke on the testing day.

### Spatial memory task

**Figure 1:**
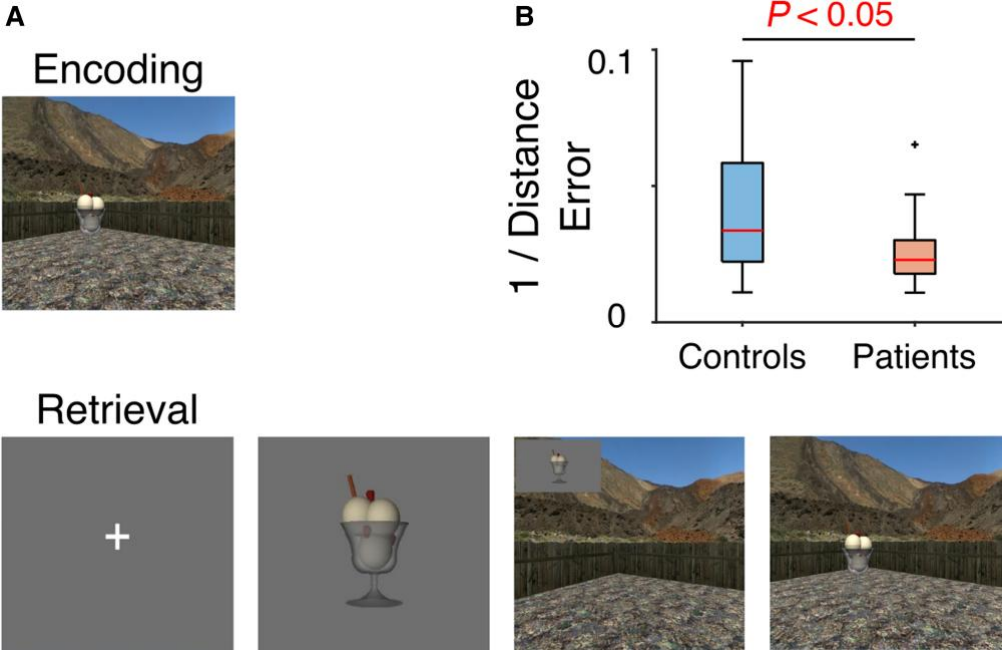
**Spatial memory task.** (**A**) Schematic. Participants navigate through the environment and make responses using a button box. During encoding, they are asked to remember the locations of four objects (one object being visible in each trial). During retrieval, a fixation cross on a grey screen is followed by an image of one object (cue period). The participants are then asked to navigate from a random start location to the retrieved location of that object and make a response. During navigation, the object image remains visible in the top left corner of the screen. Following a response, the object appears in its correct location to provide feedback. The next trial begins when the participants collide with the object. (**B**) Performance, quantified as the inverse of the average distance between remembered and actual object locations, for controls and patients. Each red line indicates the median, box edges the 25th and 75th percentiles, whiskers extend to the most extreme data points not considered to be outliers (defined as values more than 1.5 times above or below the 75th and 25th percentiles, respectively), and outliers are plotted individually. Spatial memory accuracy was significantly higher in the control group.

Inside the MEG scanner, participants performed a spatial memory task in a virtual reality environment^43^ constructed using the Unity game engine (Unity Technologies Ltd). During the task, participants navigated freely around up to three different virtual reality environments and were asked to learn—and subsequently recall—the locations of four different objects in each environment (Fig. 1A). Movement was directed using three buttons controlling left and right rotation and forward translation (via rapid acceleration to a fixed maximum speed). The environments were 100 virtual metre (vm) square arenas delineated by a solid boundary and surrounded by distant landmarks. Each environment was distinguished by the surface textures used for the floor and boundary, the location and identity of distal cues and the location and identity of the objects being memorized. At the start of each block (in each different environment), participants were placed in the centre of the environment facing in the same direction (north).

During encoding, one of four objects was visible in the environment in each trial, and participants were instructed to remember the location of that object. Once they were happy that they had remembered its location, they collided with the object to move to the next trial. There were two encoding trials for each object, in a pseudorandom order, giving eight encoding trials in each environment. Object locations were selected from 16 possible locations, so that each environment contained two objects close to the middle of the arena, one close to a corner and one near the middle of a boundary, to match difficulty across environments (with object locations not used more than once across environments).

During retrieval, each trial began with a 3 s fixation cross, followed by a 3 s cue period in which a single target object was presented on screen. Participants were then placed at a random location and orientation within the environment and asked to navigate to the location of that object and make a button press response. Participants subsequently received feedback on their performance, i.e. the cued object appeared in its correct location and the next trial began when they collided with the object. Performance in each trial was quantified using the inverse of the distance between the remembered object location and its actual location (such that larger values correspond to better performance, as used by Doeller *et al*.^38^). There were eight retrieval trials for each object, giving 32 retrieval trials in each environment. Controls and patients completed 2.70 ± 0.56 and 2.88 ± 0.33 (mean ± SD) task blocks (i.e. environments), respectively.

### MEG data collection and pre-processing

MEG data were acquired using a 275-channel axial gradiometer system (CTF Omega, VSM MedTech) at a sample rate of 480 Hz. During the recording, head position coils (attached to nasion and left and right pre-auricular sites) were used for anatomical co-registration, and eye tracking was performed using an Eyelink 1000 system (SR Research). Raw MEG data were imported into SPM12^44^ and downsampled to 200 Hz before eye blink and heartbeat artefacts were manually identified and removed using independent component analysis (ICA) implemented in FieldTrip^45^ and EEGLAB.^46^ Finally, a fifth-order, zero-phase Butterworth filter was used to remove slow drift (1 Hz high-pass) and mains noise (48–52 Hz notch) from the recordings.

Our analyses focused on periods of movement onset and complete immobility in the virtual environment. Movement onset ‘epochs’ were defined as [−3 3] s windows around the onset of continuous translational movements that lasted ≥1 s and were preceded by ≥1 s of complete immobility (consistent with previous studies^35^). This captured 25.4 ± 6.9% and 25.5 ± 6.4% of the task data for controls and patients, respectively. Stationary ‘epochs’ were defined as [−2.5 3.5] s windows around the onset of ≥2 s periods during which no translational movement occurred. This captured 51.4 ± 8.9% and 49.8 ± 7.2% of the task data for controls and patients, respectively (see Table 1 for trial numbers). Importantly, although these epochs could overlap, the overlapping time periods were not included in any of our analyses (see Supplementary Fig. 1 and further details below). Once the MEG data had been divided into movement onset and stationary epochs, artefact trials were automatically identified and removed using an underlying outlier test (with a threshold of α = 0.05).

**Table 1:** Number of movement and stationary periods (or ‘epochs’) in controls and patients

	Total movement epochs (mean ± SD)	Bad movement trials (mean ± SD, %)	Included movement trials (mean ± SD, range)	Stationary epochs (mean ± SD)	Bad stationary trials (mean ± SD, %)	Included stationary trials (mean ± SD, range)
Controls	122.6 ± 35.3	3.39 ± 4.22%	119.0 ± 36.4, 61–192	241.7 ± 75.5	3.03 ± 3.92%	234.9 ± 76.6, 110–43
Patients	142.8 ± 45.3	6.95 ± 6.89%	133.5 ± 45.8, 38–246	278.4 ± 82.5	5.91 ± 5.11%	262.9 ± 81.4, 86–408

### MEG data analysis

To examine changes in low-frequency power associated with the onset of virtual movement, we generated a time frequency spectrogram for each movement and stationary period in the 2–70 Hz range using a five-cycle Morlet wavelet transform for 40 equally logarithmically spaced frequencies. The resulting power values were log-transformed and normalized by the sum of power values across frequencies at each time point. Finally, power values were averaged across epochs for each participant, and power in the [−0.5 0.5] s window around movement onset was baseline corrected by average power in the [0 1] s window during stationary periods. Inspection of the resultant power spectrum, averaged across all participants in both groups, revealed a peak in the 4–10 Hz theta band on which subsequent analyses were focused. Source localization of 4–10 Hz theta power was performed in SPM12 using the Linearly Constrained Minimum Variance beamformer from the DAiSS toolbox, with a single-shell forward model and sources evenly distributed on a 10 mm grid co-registered to Montreal Neurological Institute (MNI) coordinates. This resulted in a set of linear weights for each participant that could generate 4–10 Hz band-pass filtered time series in source space from sensor-level data in each movement onset epoch.^47^

To look for the hexadirectional modulation of theta power, we first isolated the continuous period of translational movement following movement onset in each epoch. Next, for each task block (i.e. each virtual environment), we extracted continuous movement direction from the corresponding behavioural data and a measure of theta power by applying the Hilbert transform to band-pass filtered data in each voxel and Z-scoring the resultant time series (to match signal amplitude across voxels and participants). We then estimated grid orientation independently for each voxel using a quadrature filter^38^ applied to alternate movement onset epochs from that block. Finally, we estimated the strength of hexadirectional modulation in each voxel for the remaining movement onset epochs by linearly regressing continuous theta power against the cosine of the angular deviation from that grid orientation, with 6-fold periodicity (see Supplementary Fig. 2 for a schematic). We repeated this analysis, reversing the use of alternate epochs for estimating orientation and modulation, and averaged the regression coefficients across the two folds and then across task blocks to provide a single metric indicating the strength of hexadirectional theta modulation for each participant in each voxel. The same analysis was also performed for other rotational symmetries (specifically: 4-, 5-, 7-, and 8-fold) and hexadirectional modulation in other oscillatory bands (specifically: 2–4 Hz delta, 12–20 Hz alpha, 20–35 Hz beta and 40–70 Hz gamma). For anatomically defined region of interest analyses, we used probabilistic masks from the Julich-Brain Cytoarchitectonic Atlas^48^ thresholded at a probability value of 40%.

### Data availability

The data and custom written analysis code that support the findings of this study are freely available from https://osf.io/ght4r/.

## Results

We asked participants with a diagnosis of schizophrenia (half of whom were unmedicated) and an age-, sex- and IQ-matched control group to perform an established spatial navigation task^2,39,40,43^ using desktop virtual reality (VR) inside a MEG scanner (Fig. 1A). Consistent with previous reports,^16–20^ spatial memory performance was significantly better in the control group [*t*(38) = 2.10, *P* = 0.042, Hedge’s *g* = 0.66, confidence interval (CI) (0.028 1.32); Fig. 1B].

**Figure 2:**
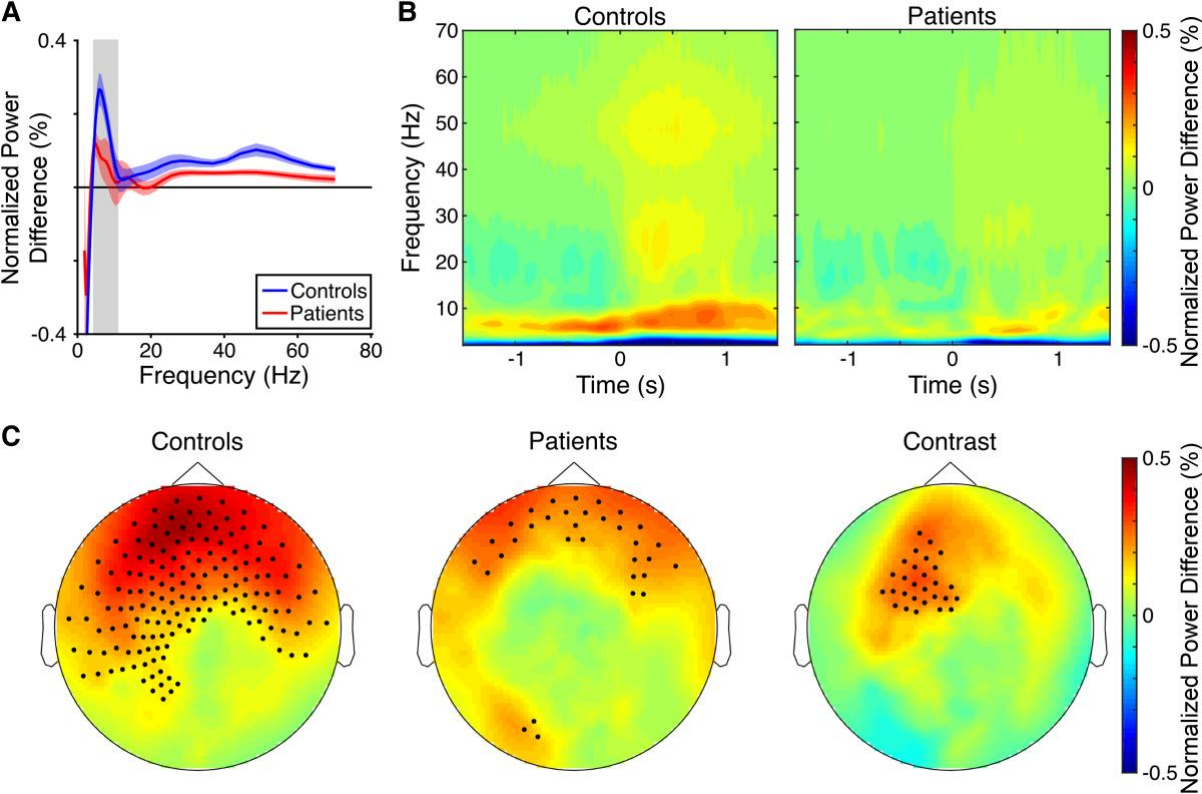
**Movement-related 4–10 Hz theta power increases in controls and patients.** (**A**) Power spectra showing normalized power during movement onset epochs (i.e. [−0.5 0.5] s around the onset of ≥1 s translational movements that were preceded by ≥1 s immobility), baseline corrected by average power during stationary periods (i.e. [0 1] s around the onset of ≥2 s periods of immobility) for controls and patients (shading indicates standard error). The grey bar delineates the 4–10 Hz theta band. (**B**) Time–frequency spectrograms showing normalized power during movement onset, baseline corrected by average power during stationary periods. Controls show a marked increase in theta power beginning ∼0.5 s prior to movement onset that is reduced in patients. (**C**) Scalp plots of normalized 4–10 Hz theta power during movement onset epochs, baseline corrected by average theta power during stationary periods for controls, patients, and for the contrast between groups. Highlighted channels show significant positive power differences at a threshold of *P* < 0.01 (uncorrected).

To look for evidence of grid-like activity during translational movement within the VR environment, we first investigated changes in oscillatory power associated with movement onset versus stationary periods. Power spectra for both groups, averaged across all sensors, showed a peak in the theta band during movement onset (Fig. 2A). Specifically, 4–10 Hz theta power was greater during movement onset than stationary periods in both controls [*t*(22) = 5.58, *P* < 0.001] and patients [*t*(16) = 2.39, *P* = 0.03], and greater in controls than patients [*t*(38) = 2.02, *P* = 0.05, *g* = 0.63, CI (0.0014 1.29); Supplementary Fig. 3A]. This is illustrated by time–frequency spectrograms of movement onset periods (Fig. 2B), which show a clear increase in theta power in the control group beginning ∼0.5 s prior to movement onset (consistent with previous reports^35,40^) that is markedly reduced in patients.

Scalp plots (showing normalized power differences between movement onset and stationary periods) illustrate that 4–10 Hz theta power increases arise over bilateral frontal and temporal sensors in both groups, with controls showing greater movement-related theta power than patients over left frontal sensors (Fig. 2C). Importantly, we found no evidence for differences in movement statistics between control and patient groups in the virtual environment that could account for these differences. Specifically, there were no differences in the average duration of movements between patients (mean ± SD = 2.29 ± 0.43 s) and controls [2.18 ± 0.5 s; *t*(38)=−0.723, *P* = 0.47] or preference to navigate close to the boundaries of the environment [patients: 79.2 ± 4.8%; controls: 79.9 ± 6.1%; *t*(38) = 0.37, *P* = 0.71], and movement speed accelerated rapidly to a fixed top speed for all participants.

**Figure 3:**
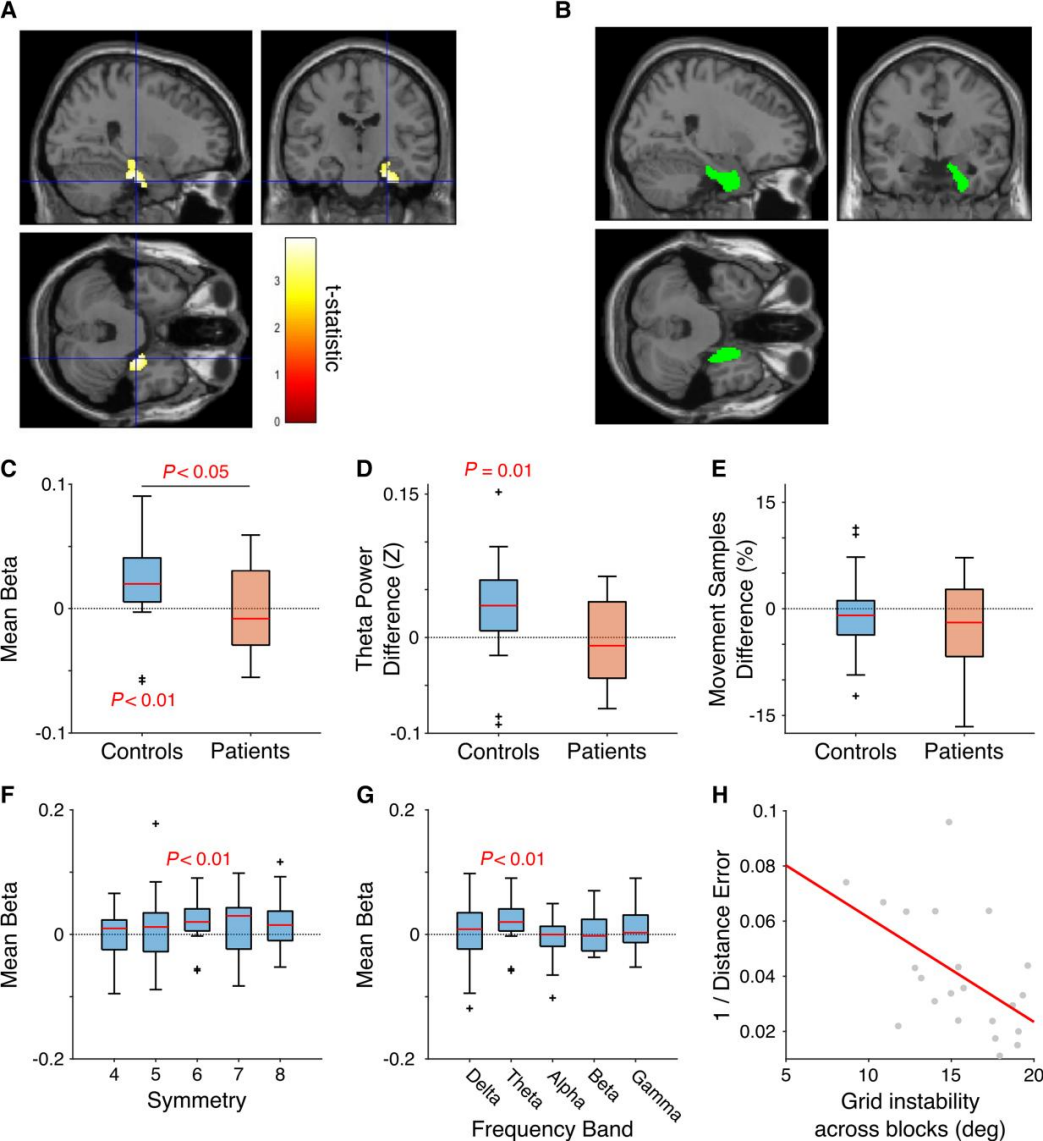
**Modulation of oscillatory power by movement direction in right entorhinal cortex.** (**A**) Regions showing significant hexadirectional modulation of 4–10 Hz theta power at the whole brain level. Only one cluster in right entorhinal cortex (peak at [18–22–44], *Z* = 4.05) passes our significance threshold of *P* < 0.05 FWE corrected (image shown at *P* < 0.005 uncorrected, for display purposes). (**B**) Image of the anatomically defined right entorhinal cortex region of interest. (**C**) Strength of hexadirectional theta modulation inside the region of interest for controls and patients, with 19/23 controls (82.6%) and 8/17 patients (47.1%) showing a positive beta coefficient. (**D**) Difference in theta power between on versus off axis movement inside the region of interest for controls and patients, with 19/23 controls (82.6%) and 7/17 patients (41.2%) showing greater on versus off axis theta power. (**E**) Difference in the percentage of movement samples that occurred during on versus off axis movement for controls and patients. (**F**) Theta modulation by 4–8-fold movement direction inside the region of interest for controls. (**G**) Strength of hexadirectional modulation of delta (2–4 Hz), theta (4–10 Hz), alpha (12–20 Hz), beta (20–35 Hz) and gamma (40–70 Hz) frequency bands inside the region of interest for controls. (**H**) Correlation between performance, quantified as the inverse of the average distance between remembered and actual object locations, and grid (in)stability across task blocks for controls. Each red line indicates the median, box edges the 25th and 75th percentiles, whiskers extend to the most extreme data points not considered to be outliers (defined as values more than 1.5 times above or below the 75th and 25th percentiles, respectively) and outliers are plotted individually.

Next, we looked for hexadirectional modulation of movement-related theta power across the whole brain using established methods^38^ (see Supplementary Fig. 2 for further details). Remarkably, the control group showed a single significant cluster of hexadirectional theta modulation in the vicinity of right entorhinal cortex (Fig. 3A). In contrast, the patient group showed no clusters that passed our threshold of *P* < 0.05 family-wise error (FWE) corrected across the whole brain.

To further characterize this effect, we extracted the strength and orientation of hexadirectional theta power modulation from each voxel in an anatomically defined right entorhinal region of interest for each participant (Fig. 3B). Consistent with the whole brain results, this revealed significant hexadirectional modulation of 4–10 Hz theta power for controls [*t*(22) = 3.04, *P* = 0.0059] but not patients [*t*(16)=−0.04, *P* = 0.97], and significantly stronger hexadirectional modulation for controls than patients [*t*(38) = 2.08, *P* = 0.044, *g* = 0.65, CI (0.02 1.31); Fig. 3C]. Similarly, theta power in this region of interest was greater during movement aligned versus misaligned with the grid axes for controls [(i.e. within ±15° of the fitted grid orientation versus other movement directions; *t*(22) = 2.82, *P* = 0.01; Fig. 3D], despite no difference in the proportion of movement samples with aligned versus misaligned directions [*t*(22)=−0.70, *P* = 0.49; Fig. 3E]. Importantly, theta power from this region of interest was not significantly modulated by 4-, 5-, 7- or 8-fold movement direction in the control group, although we note a trend towards significance for 8-fold modulation [*t*(22) = 2.03, *P* = 0.055; all others *P* > 0.27; Fig. 3F], nor was there any evidence for hexadirectional modulation of delta, alpha, beta, or gamma frequency band activity in this region (all *P* > 0.26; Fig. 3G). In addition, we found no evidence for the hexadirectional modulation of theta power within a corresponding anatomically defined left entorhinal region of interest (Supplementary Fig. 3B).

Reassuringly, grid orientation across voxels inside the right entorhinal region of interest (within each task block and data partition) was more consistent than expected by chance [5.33 ± 2.25°, chance = 15°; *t*(22)=−20.7, *P* < 0.001], as was grid orientation across data partitions, each including half of the trials [within each task block and region of interest voxel; 12.9 ± 3.55°; *t*(22)=−2.85, *P* = 0.0093]. However, grid orientation across blocks (within each data partition and voxel inside the region of interest) was no more consistent than expected by chance [[15.5 ± 3.05°, *t*(21) = 0.71, *P* = 0.49], suggesting that grid patterns randomly realigned with the visually distinct square environment encountered in each task block. Importantly, we found no evidence for a relationship between theta power during movement onset (averaged across all sensors) and the strength of hexadirectional modulation inside the region of interest (Pearson’s *r* = 0.32, *P* = 0.14); or between theta power during movement onset (averaged across all voxels within the region of interest) and the strength of hexadirectional modulation in the same region (*r* = 0.25, *P* = 0.25). This suggests that differences in the magnitude of hexadirectional modulation across participants did not arise simply from differences in the power of the underlying theta oscillation.

Finally, we looked for a relationship between the hexadirectional modulation of 4–10 Hz theta power inside the region of interest and our behavioural data. Although we found no evidence for a correlation between the strength of hexadirectional modulation and task performance across controls (*r* = 0.15, *P* = 0.49), we did find a significant relationship between the consistency of the grid orientation across blocks and task performance (*r* = −0.52, *P* = 0.013; Fig. 3H). This indicates that control participants with grid patterns that were more consistent across task blocks tended to more accurately remember object locations in the VR environments. Within the patient group, we found no evidence for differences in task performance, medication or symptom severity between participants with (8/17) and without (9/17) hexadirectional modulation of theta power in the same region of interest (all *P* > 0.22).

## Discussion

Our results demonstrate that people with schizophrenia show worse spatial memory and less movement-related theta power during a virtual spatial navigation task than a matched control group. They also lack the hexadirectional modulation of theta power in right entorhinal cortex observed in the control group, which is consistent with the presence of stable grid cell firing patterns. Importantly, the stability of grid orientation across task blocks in the control population correlated positively with their performance in the spatial memory task, suggesting a functional relationship between grid firing patterns and spatial memory. This is the first demonstration of hexadirectional theta modulation in MEG, building on previous studies showing similar patterns in BOLD signal throughout the default mode network,^27,38^ in high-frequency activity from the anterior temporal lobe in both MEG and intracranial EEG recordings^49^ and in entorhinal theta power from intracranial EEG recordings.^36,37^ Crucially, however, the relationship between grid cell activity at the neural level, network level modulations of theta or high-frequency power in the local field potential or in MEG and the BOLD signal measured using functional MRI (fMRI) is not clear, and merits further attention.

Previous studies have reported impaired spatial navigation associated with hippocampal anomalies in schizophrenia.^16–20^ In particular, people with schizophrenia are selectively impaired in spatial navigation strategies based on cognitive mapping, rather than single-landmark (response-based) strategies.^19,20^ Schizophrenia is also associated with impairments in associative inference and acquisition of relational knowledge,^2,50–52^ in which the hippocampal formation—and grid cells in particular—are thought to play a key role.^28^ Our findings therefore suggest that dysfunctional grid coding may underlie atypical inference and poor acquisition of relational knowledge in schizophrenia. Grid firing patterns may be supported by attractor network dynamics,^53^ and attractor states are thought to be more unstable in schizophrenia,^54,55^ potentially due to reduced α5-GABA-A receptor density in the MTL.^56^ We speculate that this may increase reliance on striatal learning mechanisms, making inferences more dependent on individual landmarks (or, perhaps, events) than structured relational knowledge of the world.

We note that movement-related increases in theta power and the hexadirectional modulation of theta power by movement direction appear to be related but distinct phenomena. First, we found no correlation between movement-related theta power and the strength of hexadirectional modulation across our control group. Second, movement-related theta power increases at the sensor level are most prominent over left frontal regions, while the hexadirectional modulation of theta power by movement direction is restricted to right entorhinal cortex. In contrast, we found no evidence for hexadirectional theta modulation in an anatomically defined left entorhinal cortex region of interest, although we are reluctant to over-interpret this absence of evidence, given previous observations of grid cells^22^ and the hexadirectional modulation of theta power in bilateral entorhinal cortex.^36,37^

In summary, in healthy volunteers performing a virtual spatial navigation task, we have shown grid-like modulation of MEG theta power localized to the right entorhinal cortex whose consistency of orientation across virtual environments correlates with spatial memory performance. Relative to this baseline, we have shown that people with a diagnosis of schizophrenia have impaired spatial memory performance, reduced movement-related theta oscillations and disrupted grid-like modulation of theta power. This extends previous work showing structural and functional impairment of the hippocampal formation in schizophrenia and selective deficits of hippocampus-dependent strategies in spatial navigation. Future studies could address a possible role of grid cell populations in impaired structural knowledge and inference in schizophrenia.

## Supplementary Material

awac416_Supplementary_Data
