# Supplementary material for: Reduced grid-like theta modulation in schizophrenia
Source: Brain. 2022 Nov 10;146(5):2191–8. doi: 10.1093/brain/awac416 (PMC10151182; doi:10.1093/brain/awac416)
Supplement: awac416_Supplementary_Data [file awac416_supplementary_data.pdf]

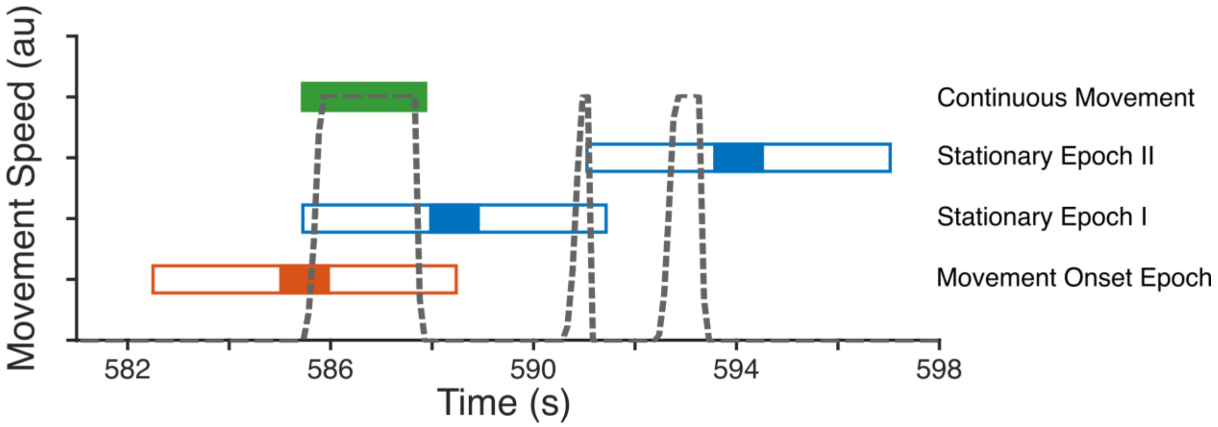

Figure S1: Example time course of task conditions. Analyses of movement related changes in oscillatory power (shown in Fig. 2 and Supplementary Fig. 3A) compare [-0.5 0.5] s windows around the onset of movements that last ≥1 s and are preceded by ≥1 s stationary periods (‘movement onset epoch’, shaded red box) with [0 1] s windows around the onset of stationary periods which last ≥2 s (‘stationary epochs’, shaded blue boxes). In both cases, the data are extracted from wider 6 s ‘epochs’ (unfilled coloured boxes) that can overlap, and provide padding to avoid edge effects in signal processing. Importantly, however, the time windows of interest are always separated by ≥0.5 s due to the duration thresholds for movement and stationary periods described above. Analyses of oscillatory power modulation by movement direction (shown in Fig. 3 and Supplementary Fig. 3B) focus on the full period of translational movement following movement onset in each 6 s epoch (‘continuous movement’, shaded green box). All other task periods (i.e. including movement or stationary periods that do not meet our duration thresholds, stationary periods from 1 s after movement cessation to 0.5s before movement onset, and movement periods from 3 s after movement onset) are unused.

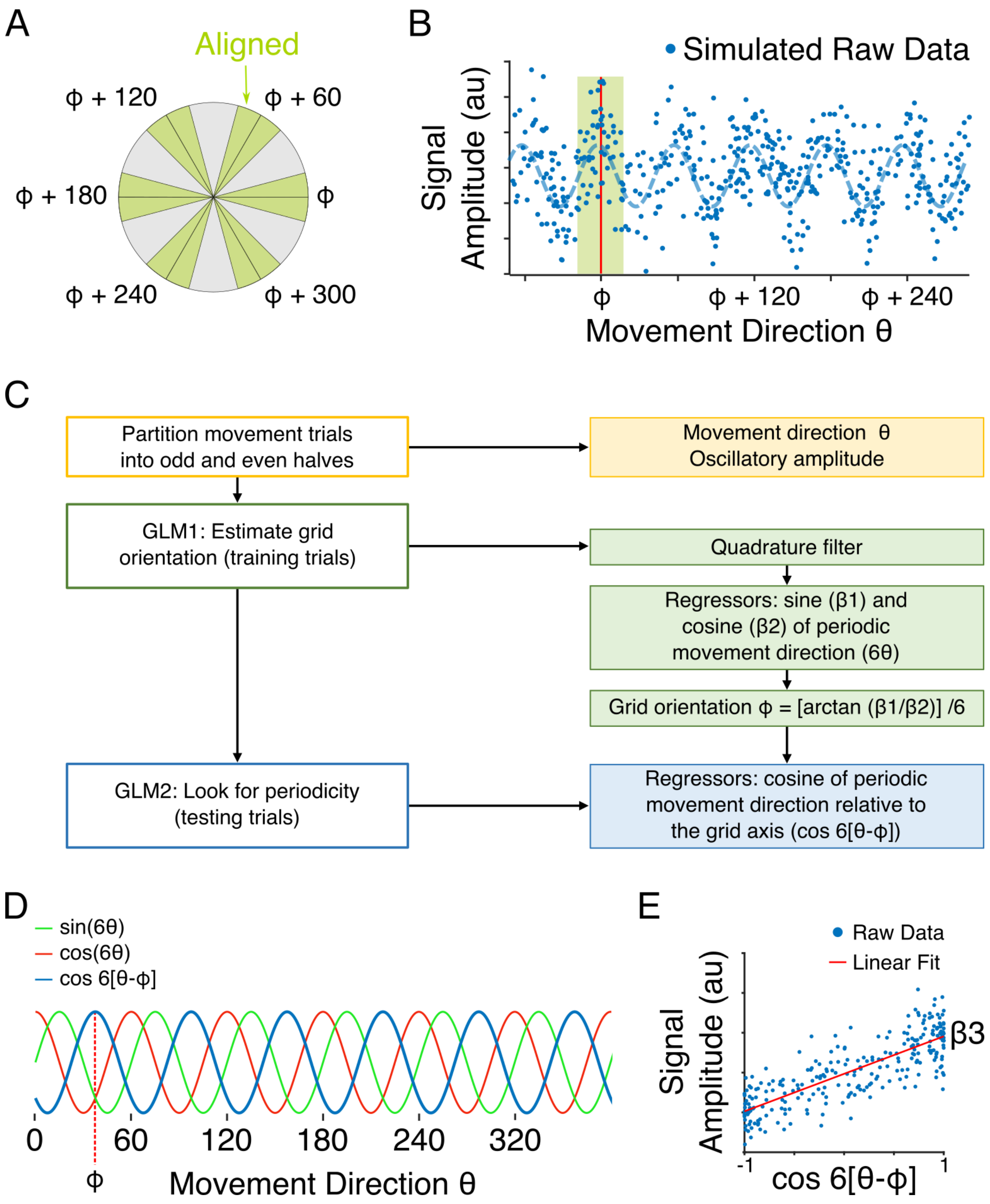

Figure S2: Identifying hexadirectional modulation of theta band activity in MEG. **A)** Each movement direction in the virtual environment is either aligned (green) or misaligned (grey) with the orientation φ of an underlying grid firing pattern. **B)** We hypothesise that the amplitude of theta band activity in the MEG signal is sinusoidally modulated by movement direction θ with six-fold (hexadirectional) rotational symmetry. **C)** To test this hypothesis, we first partitioned movement epochs into alternate even and odd trials. For each movement epoch, we

then extracted the direction of movement in the VR environment θ and power in the theta band at each time step. In half of the movement epochs, we used a quadrature filter to estimate the orientation of the underlying grid. Specifically, we calculated the sine and cosine of movement direction θ with 6-fold periodicity and fit those $\cos(6\theta)$ and $\sin(6\theta)$ regressors to oscillatory power in a first GLM (GLM1). This produced regressor coefficients $\beta 1$ and $\beta 2$ which could be used to estimate grid orientation $\varphi = [\arctan(\beta 2/\beta 1)] / 6$. We then tested whether the amplitude of oscillatory power in the other half of movements epochs was sinusoidally modulated at this orientation. Specifically, we fit the cosine of movement direction with 6-fold periodicity and orientation φ - $\cos(6\theta-\varphi)$ - to oscillatory power in a second GLM (GLM2). This produced a regressor coefficient $\beta 3$ which characterises the strength of hexadirectional modulation. **D)** Graphic representation of the $\cos(6\theta)$ and $\sin(6\theta)$ regressors used in GLM1; and $\cos(6\theta-\varphi)$ regressor used in GLM2. **E)** Simulated data are used to show the linear fit between $\cos(6\theta-\varphi)$ and oscillatory power in GLM2. The slope of the linear fit corresponds to the strength of hexadirectional modulation. Panels A and D adapted from [37].

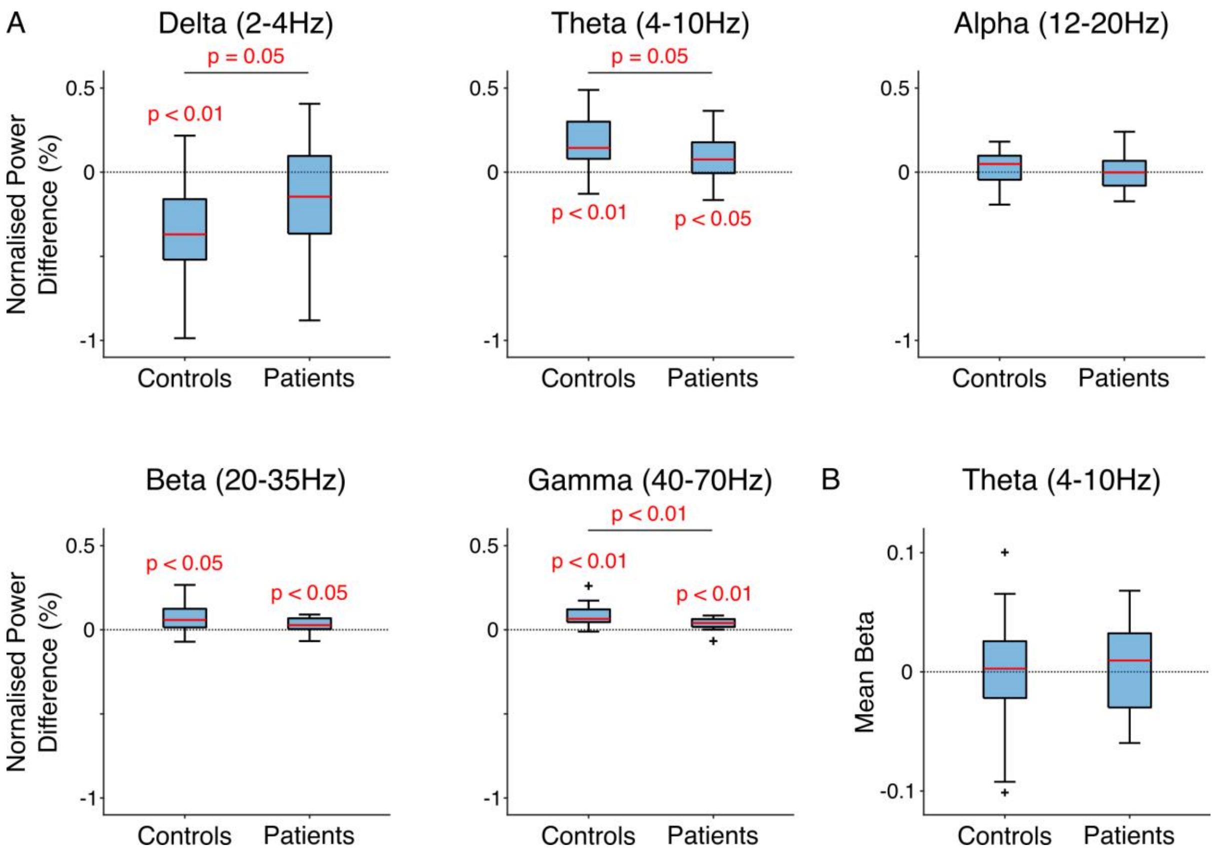

Figure S3: Movement related changes in oscillatory power and hexadirectional modulation of theta power in left entorhinal cortex. **A**) Normalised oscillatory power during movement onset epochs (i.e. [-0.5 0.5] s around the onset of ≥1 s translational movements that were preceded by ≥1 s immobility), baseline corrected by average power during stationary periods (i.e. [0 1] s around the onset of ≥2 s periods of immobility) for controls (in blue) and patients (in red) in the delta (2-4Hz), theta (4-10Hz), alpha (12-20Hz), beta (20-35Hz) and gamma (40-70Hz) frequency bands, averaged across all sensors. In addition to the significant changes in theta power described in the main text, we find evidence for movement related decreases in delta power in both controls ($t(22)=-5.93$, $p<0.001$) and patients ($t(16)=-2.11$, $p=0.05$), as well as a significant difference between groups ($t(38)=2.02$, $p=0.05$, $g=0.633$, *CI* [0.0024 1.29]); movement related increases in beta power in both controls ($t(22)=3.74$, $p=0.001$) and patients ($t(16)=2.76$, $p=0.014$), with no difference between groups ($t(38)=1.57$, $p=0.13$); and movement related increases in gamma power in both controls ($t(22)=6.39$, $p<0.001$) and patients ($t(16)=4.12$, $p<0.001$), as well as a significant difference between groups ($t(38)=2.74$, $p=0.0092$, $g=0.86$, CI [0.221 1.54]); but no changes in alpha power in either group (both $p>0.15$) or difference between groups ($t(38)=0.557$, $p=0.58$). **B)** Absence of hexadirectional theta modulation inside an anatomically-defined left entorhinal ROI for controls ($t(22)=-0.184$, $p=0.856$) and patients ($t(16)=0.45$, $p=0.659$). There is no significant difference in the strength

of hexadirectional modulation between groups in this ROI ($t(38)=-0.419$, $p=0.677$). Each red line indicates the median, box edges the 25th and 75th percentiles, whiskers extend to the most extreme datapoints not considered to be outliers (defined as values more than 1.5 times above or below the 75th and 25th percentile, respectively), and outliers are plotted individually.
